# Detección de especies de *Helicobacter* en humanos y sus perros

**DOI:** 10.31053/1853.0605.v80.n3.36769

**Published:** 2023-09-29

**Authors:** Corina Guendulain, Pablo Tamiozzo, Griselda González, Marina Caffaratti

**Affiliations:** 1 Universidad Nacional de Río Cuarto. Facultad de Agronomía y Veterinaria. Departamento Clínica Animal. Clínica de Pequeños Animales; 2 Universidad Nacional de Río Cuarto. Facultad de Agronomía y Veterinaria. Departamento Patología Animal. Área de Biología Molecular. Cátedra de Enfermedades Transmisibles y Tóxicas de los Porcino.; 3 Universidad Nacional de Río Cuarto. Facultad de Agronomía y Veterinaria. Departamento Clínica Animal. Clínica de Pequeños Animales

**Keywords:** Helicobacter, estómago, salud pública veterinaria, Helicobacter, estômago, saúde pública veterinária, Helicobacter, stomach, veterinary public health

## Abstract

*Helicobacter* es un género bacteriano gram negativo que coloniza el estómago de humanos y perros, entre otros mamíferos. En la mucosa gástrica del hombre la especie más frecuentemente hallada es *H. pylori*, sin embargo, puede albergar otras especies, como *H. suis*, *H. bizzozeronii, H. felis, H. salomonis* y *H. heilmannii sensu stricto*, denominadas *Helicobacter* no *H. pylori* (NHPH), algunas de las cuales colonizan también el estómago del perro. La detección de especies NHPH en la mucosa del estómago de seres humanos con enfermedad gástrica y en un alto porcentaje de perros como mascotas, sugiere la posibilidad de que estos animales jueguen un rol importante en la patogenia y transmisión de la infección al hombre, postulándose la vía de transmisión oral-oral o bien fecal-oral. El objetivo de este trabajo fue determinar si individuos con signos de gastritis crónica que tienen *Helicobacter* spp. y que poseen perros como mascotas, comparten con ellos las mismas especies. Para ello se estudió por PCR la presencia de *Helicobacter* spp. en muestras gástricas de 30 pacientes con signos clínicos de gastritis crónica y diagnóstico histopatológico de infección gástrica con *Helicobacter* spp. También se estudió la presencia de estas bacterias en sus perros para determinar si existe correspondencia en las especies presentes en ambos. La única especie encontrada en humanos fue *H. pylori*, mientras que en los perros se encontraron *H. bizzozeronii*, *H. felis*, *H. salomonis* y *H. heilmannii*. En este trabajo no se encontraron las mismas especies en los perros y en sus propietarios.

## Abstract (en)

*Helicobacter* is a genus of gram-negative bacteria that colonizes the stomach of humans and dogs, among other mammals. *H. pylori* is the most frequently found species in the human gastric mucosa, however, there are other species of *Helicobacter* in the stomach of humans, such as *H. suis*, *H. bizzozeronii, H. felis, H. salomonis* and *H. heilmannii* sensu stricto, called non *H. pylori Helicobacter* (NHPH) some of which also colonize the dog's stomach. The detection of NHPH in the stomach mucosa of humans with gastric disease, and in a high percentage of dogs as pets, suggests the possibility that these animals play an important role in the pathogenesis and transmission of infection to man, postulating the path of transmission oral-oral or fecal-oral. The aim of this study was to determine whether people with signs of chronic gastritis who have *Helicobacter* spp. and who own dogs as pets, share the same species with them. The presence of *Helicobacter* spp. in gastric samples of 30 patients with clinical signs of chronic gastritis and histopathological diagnosis of gastric infection with *Helicobacter* spp. was analyzed by PCR. The presence of *Helicobacter* spp. in their dogs was also studied, in order to determine correspondence of *Helicobacter* species present in both. In human gastric samples, *H. pylori* was the only species found, while in dogs, samples were positive to *H. bizzozeronii, H. felis, H. salomonis* and *H. heilmannii*. In this work the same species were not found in dogs and their owners.

## Resumo

Introdução: *Helicobacter* é um gênero de bactéria gram negativa que coloniza o estômago de humanos e cães, entre outros mamíferos. Na mucosa gástrica do homem a espécie mais freqüentemente encontrada é *H. pylori*, porém, pode abrigar outras espécies, como *H. suis, H. bizzozeronii, H. felis, H. salomonis* e *H. heilmannii* sensu stricto, denominado *Helicobacter sem H. pylori* (NHPH), alguns dos quais também colonizam o estômago do cão. A detecção de espécies de NHPH na mucosa do estômago de seres humanos com doença gástrica e em alta porcentagem de cães como animais de estimação, sugere a possibilidade de que esses animais desempenhem um papel importante na patogênese e transmissão da infecção ao homem, postulando a via oral transmissão oral ou fecal-oral. O objetivo deste trabalho foi determinar se indivíduos com sinais de gastrite crônica portadoras de *Helicobacter* spp. e que possuem cães como animais de estimação, compartilham a mesma espécie com eles. Para isso, a presença de *Helicobacter* spp., foi estudada por PCR em amostras gástricas de 30 pacientes com sinais clínicos de gastrite crônica e diagnóstico histopatológico de infecção gástrica por *Helicobacter* spp. A presença dessas bactérias em seus cães também foi estudado para determinar se há correspondência nas espécies presentes em ambos. A única espécie encontrada em humanos foi *H. pylori*, enquanto *H. bizzozeronii*, *H. felis*, *H. salomonis* e *H. heilmannii* foram encontrados em cães. Neste trabalho, a mesma espécie não foi encontrada em cães e seus donos.

**CONCEPTOS CLAVE**

*Helicobacter pylori* es causa frecuente de gastritis crónica, sin embargo, también pueden encontrarse en la mucosa gástrica otras especies de Helicobacter, como *H. suis*, *H. bizzozeronii*, *H. felis*, *H. salomonis* y *H. heilmannii* *sensu stricto*, denominadas Helicobacter no *Helicobacter pylori* (NHPH). Estas bacterias NHPH también habitan la mucosa gástrica de la mayoría de los perros, sin embargo, se desconoce si existe transmisión zoonótica. Si bien la presencia de bacterias NHPH ha sido estudiada tanto en perros como en personas por investigadores de distintos países y, además, se registran algunos reportes del hallazgo de individuos y sus perros infectados simultáneamente, no existen estudios sobre la presencia de NHPH en un número significativo de humanos y de sus perros. En nuestro país, se reporta un único estudio de las especies de NHPH en perros (Guendulain et al., 2016), pero no en humanos. Por lo tanto esta investigación aporta información inédita acerca de estas bacterias en nuestro país, tanto en personas como en perros.

**Divulgación**

Es frecuente encontrar en las personas que tienen síntomas de gastritis crónica, como acidez, sensación de llenado estomacal, náuseas, etc., una bacteria en su mucosa gástrica llamada *Helicobacter pylori*. Los perros, en su mayoría, también son portadores de bacterias de este género, pero las especies encontradas son otras, diferentes a *H. pylori*, denominadas Helicobacter no *H. pylori* (NHPH). La presencia de estas bacterias en la mucosa del estómago de seres humanos con enfermedad gástrica y en un alto porcentaje de perros, sugiere la posibilidad de que estos animales tengan alguna implicancia en la transmisión de la infección al hombre. El objetivo de este trabajo fue determinar si las personas con signos de gastritis crónica que tienen *Helicobacter* spp. y que poseen perros como mascotas, comparten con ellos las mismas especies.

## Introducción

*Helicobacter* (*H*) es un género bacteriano gram negativo que coloniza el estómago de perros y humanos ^1 2^ . Estas bacterias tienen forma bacilar o espiralada, aunque en condiciones ambientales desfavorables pueden modificar su morfología a cocoide, un estado viable pero no cultivable que les permite permanecer latentes durante mucho tiempo ^3^ .

La especie más frecuentemente hallada en la mucosa gástrica del hombre es *H. pylori*, sin embargo, aunque con menor prevalencia, pueden encontrarse otras especies de forma espiralada y de mayor tamaño que *H. pylori* denominadas *Helicobacter* no *H. pylori* (NHPH) ^4^ . De estas especies, la más prevalente es *H. suis* ^1^ , pero también se ha reportado el hallazgo de *H. salomonis*, *H. bizzozeronii*, *H. felis* y *H. heilmannii sensu stricto*. La prevalencia de infección por especies NHPH en humanos varía entre 0,2 y 6%, según la zona geográfica ^5^ , mientras que *H. pylori* es encontrado en el 30 al 100% de las gastroscopías ^6^ .

En el perro las bacterias NHPH gástricas tienen una prevalencia que varía entre 67 a 86% en aquellos clínicamente sanos y entre 61 a 100% en perros con vómito crónico, y es común la infección de dos o más especies en forma conjunta ^7-11^ . Si bien hay consenso en que *H. pylori* no infecta de forma natural al perro, en Austria ^12^ , en Chile ^13^ y en Turquía ^14^ se encontró esta especie en unos pocos perros.

La detección de NHPH en seres humanos y en sus perros, sugiere la posibilidad de que estos animales jueguen un rol en la transmisión de la infección al hombre, considerándose de riesgo zoonótico. Tal es el caso de una niña en la que se hallaron bacterias NHPH similares en morfología a las que se encontraron en su perro ^15^ y el de una mujer con úlcera péptica en la que se evidenció la presencia de *H. felis*, mientras que en su perro se encontró *H. felis*, *H. bizzozeronii* y " *Candidatus H. heilmannii"* ^16^ .

En nuestro país, sólo hay antecedentes bibliográficos acerca del estudio de las especies de NHPH en perros ^17^ , pero no en humanos.

En este contexto, el objetivo de este trabajo fue determinar si pacientes con signos y síntomas de gastritis crónica que tienen *Helicobacter* spp. y que poseen perros como mascotas, comparten las mismas especies con estos.

## Materiales y métodos

Se realizó un estudio cualitativo, en el cual se incorporaron los individuos que cumplieran con los siguientes criterios: pacientes humanos atendidos en el Nuevo Hospital de Río Cuarto con signos y síntomas clínicos de gastritis crónica, los cuales tenían perros como mascotas y resultado positivo a formas compatibles con *Helicobacter* spp. en la histopatología de sus biopsias gástricas (n=30).

Reparo ético: los pacientes prestaron su conformidad por escrito para la participación personal y la de sus mascotas en el estudio. Se siguieron las pautas éticas internacionales para la investigación biomédica, propuestas por el Consejo de Organizaciones Internacionales de las Ciencias Médicas (CIOMS) en colaboración con la OMS. Esta investigación fue aprobada por el Comité Institucional de Ética en Investigación en Salud de la UNRC (CIEIS UNRC).

Las muestras de la mucosa gástrica de los pacientes y de sus perros para la realización de la técnica de PCR se obtuvieron mediante endoscopía. La extracción del ADN de cada una de las muestras fue realizada con *kit* comercial (DNAzol, Invitrogen, CA, USA) de acuerdo con las instrucciones del fabricante, y fue almacenado a -20 ºC hasta su procesamiento.

Con el ADN obtenido se realizó primero la PCR para determinar la presencia del género *Helicobacter* ^18^ , amplificando un fragmento de 780 pb del gen *16S ARNr* y utilizando cebadores género específicos previamente publicados ^19^ . Luego, tanto a las muestras positivas como a las negativas, se les realizaron tres PCR: una para detectar *H. pylori* ^20, 21^ , una PCR múltiple para detectar *H. bizzozeronii, H. felis* y *H. salomonis* ^22^ y otra para detectar *H. suis* ^23^ . Finalmente, se realizó una PCR que detecta el gen *UreAB* y que permite identificar especies por secuenciación ^24^ . Como controles positivos se utilizó ADN de cepas de referencia ^22^ .

Las amplificaciones se realizaron con un ciclador Labnet MultiGene™ Gradient PCR Thermal Cycler, Edison, EUA. En todos los casos el producto de la PCR fue corrido en un gel de agarosa al 1,5%, teñido con bromuro de etidio Inbio Highway® Argentina (5 ugrs/ml), en 1x de buffer TAE, a 70 V durante 30 min, y observado bajo luz UV. Los productos de amplificación del ADN fueron purificados (Puriprep-GP Kit, Inbio Highway, Argentina), cuantificados y secuenciados (ABI 3130xl; Applied Biosystems, Instituto de Biotecnología INTA-Castelar) con los cebadores descriptos. Las secuencias obtenidas fueron visualizadas con el software BioEdit y alineadas y comparadas con las disponibles en la base de datos GENBANK mediante el software BLAST (http://www.ncbi.nlm.nih.gov/BLAST/).

Se realizó un análisis exploratorio de datos, y para relacionar las especies de bacterias encontradas se realizó una regresión logística. Todos los análisis fueron realizados con el software R ^25^ .

## Resultados

En la tabla I se muestran los resultados de las PCR realizadas a partir de las muestras de los individuos estudiados. En humanos, cabe aclarar que hubo una muestra que, a pesar de haber resultado negativa a la PCR género específica (*Helicobacter* spp.), resultó positiva a la PCR *H. pylori* específica, por lo cual la detección de positivos para *H. pylori* (25/30) fue mayor que para *Helicobacter* spp (24/30). En perros, de manera similar, una muestra que había resultado negativa en la PCR género específica (*Helicobacter* spp.), resultó positiva en la PCR especie específica de *H. felis*, por lo que en total se detectaron 24 individuos portadores de especies de *Helicobacter*.

**Tabla N° 1:** Especies de *Helicobacter* encontradas en las biopsias gástricas de humanos y perros determinadas por PCR.

*Helicobacter* spp.	*H. pylori*	*H. bizzozeronii*	*H. felis*	*H. salomonis*	*H. heilmannii*	*H. cynogastricus*	*H. suis*
24/30	25/30	0/30	0/30	0/30	0/30	0/30	0/30
80%		83,3%	0%	0%	0%	0%	0%	0%

La identidad de las especies fue determinada en 11 perros (45,8%), siendo uno de ellos portador de más de una especie (*H. bizzozeronii* y *H. salomonis*).

*H. bizzozeronii* y *H. felis* pudieron determinarse a través de la PCR múltiple, mientras que mediante la PCR basada en el gen ureAB se identificaron *H. felis* (BankIt2672097 P4 OQ418021), *H. salomonis* (BankIt2672093 P2 OQ418020 y BankIt2672098 P5 OQ418022) y *H. heilmannii* (BankIt2672103 P OQ418023).

En la regresión logística se obtuvo un valor de OR de 5 con un IC95% (0,73-34,34) - (p=0,10).

## Discusión

En humanos, la prevalencia hallada mediante PCR a nivel de género fue del 80% (24/30), mientras que para *H. pylori* fue del 83,3% (25/30), ya que hubo un paciente negativo en la PCR para *Helicobacter* spp. pero positivo mediante el ensayo específico para *H. pylori*, y no se detectaron resultados positivos para las restantes especies bacterianas estudiadas. Esto coincide con los datos aportados por la bibliografía en cuanto a que la especie que predomina en la mucosa gástrica del humano es *H. pylori*, encontrándose en el 30 al 100% de las gastroscopías ^2^ , y que la prevalencia de infección por bacterias NHPH, aunque varía según la zona geográfica, es mucho más baja ^5^ . A su vez, en cuanto a la prevalencia de las distintas especies de NHPH, la de *H. suis* es más alta que la de otras especies, y siguen en orden decreciente *H. salomonis*, *H. felis* y *H. bizzozeronii* ^8^ .

Dado que la bibliografía informa la posible condición zoonótica de las especies NHPH, y teniendo en cuenta la relación estrecha que existía entre las personas estudiadas y sus perros, era esperable encontrar algún humano infectado con estas especies. Sin embargo, no se detectó ninguna de las bacterias NHPH testeadas en ninguno de los individuos humanos incluidos en nuestro estudio. Esto podría deberse a que estas bacterias no estén tan adaptadas al ambiente gástrico humano, ya que la infección de los seres humanos con estas bacterias es rara a pesar del contacto frecuente con los animales domésticos, mientras que *H. pylori* podría estar mejor adaptado a los humanos y no a los perros. Además, como las infecciones concomitantes de *H. pylori* y especies NHPH son escasamente descriptas, se ha sugerido que *H. pylori* podría inhibir el crecimiento de las NHPH y viceversa ^26^ . No obstante, se debe tomar en cuenta la posibilidad de un subregistro, debido a que las infecciones con bacterias NHPH producen signos clínicos más leves que las infecciones con *H. pylori*, por lo cual probablemente las personas con dicha infección no concurren a la consulta médica y, por lo tanto, quedan subdiagnosticadas. Solamente un bajo porcentaje de pacientes humanos con gastritis severa tienen, a la observación microscópica de biopsias gástricas, bacterias grandes, de forma espiralada, compatibles con NHPH ^5^ .

Por otra parte, la prevalencia de especies NHPH encontrada en perros mediante PCR fue del 80% (24/30), similar a lo reportado por otros investigadores ^8, 10-11^ , a excepción de Amorim y col. ^9^ , quienes detectaron bacterias NHPH sólo en el 47,8% de los perros.

La identidad de las especies fue determinada en el 45,8% (11/24) de los perros positivos a la presencia de *Helicobacter* spp.; en el resto (54,2%) no se pudo determinar la especie mediante las PCR especie específicas, probablemente por no tratarse de alguna de las especies estudiadas. Aunque en el estudio de Wiinberg y col. ^27^ se logró identificar las especies en el 82% de los caninos positivos, existen otros trabajos que reportan que en más del 50% de las muestras positivas de perros no se logra identificar la especie ^28 10^ .

En este estudio no se encontró *H. pylori* en las muestras de perros, en coincidencia con un reporte previo reciente ^11^ . Sin embargo, hay evidencia sobre la presencia de esta bacteria en perros; Buczolits y col. ^12^ secuenciaron parcialmente el gen 16S ARNr en muestras gástricas de 2 perros, aunque no lograron su aislamiento. Los autores sugirieron que quizás la mucosa gástrica canina no permite la supervivencia de *H. pylori*, lo que podría inducir el cambio de su forma espiralada a cocoide. Esto podría explicar que no se haya podido realizar su aislamiento, ya que estas formas cocoides son viables, pero no cultivables. Otras publicaciones también dan cuenta de la presencia de *H. pylori* en pocos perros ^13 14^ , aunque se plantea la posibilidad de que se trate de una especie muy estrechamente relacionada, con moderada similitud a *H. pylori*, con 96.6%-98% de identidad genética. En conjunto, los resultados no permiten descartar la hipótesis sobre la posible transmisión zoonótica de *H. pylori* a partir de perros, siendo por tanto una cuestión que aún debe investigarse.

En este estudio *H. bizzozeronii* fue la especie más prevalente en perros, coincidiendo con otras publicaciones ^7,22, 28^ . En cambio, otros grupos de investigadores encontraron como especie más prevalente a *H. heilmannii* ^9 29^ , aunque es importante destacar que uno de ellos sólo estudió dos especies, *H. heilmannii* y *H. felis*, y el otro encontró que los amplicones obtenidos en la PCR específica para *H. heilmannii* solamente mostraron un 92% de homología con *H. heilmannii*, por lo que fueron reclasificados como *H. heilmannii-like*. Estas especies podrían ser una variante de *H. heilmannii* adaptada al huésped o ser una nueva especie. Por otro lado, si bien algunos autores ^27^ encontraron a *H. bizzozeronii* como la especie más prevalente, destacaron que *H. heilmannii* fue la segunda especie más frecuentemente hallada. Incluso, en una investigación más reciente predominó *H. heilmannii* sobre las demás especies ^11^ .

*H. felis* se encontró en un número limitado de perros, en concordancia con otros investigadores ^7,9,11, 29^ , sin embargo hubo un estudio en el cual se diagnosticó esta especie en más del 50% de los perros ^8^ .

*H. salomonis* también se encontró en pocos perros, como han comunicado otros investigadores ^8 11^ . Sin embargo, hay un reporte donde *H. salomonis* aparece como la segunda especie más prevalente ^9^ . Este hallazgo cobra importancia, dado que esta es la segunda especie de NHPH, en orden de frecuencia de presentación en las personas, luego de *H. suis* ^8^ .

Una de las muestras presentó 100% de similitud con una secuencia recientemente publicada, pero sin identidad de especie, de *Helicobacter* no cultivable recuperado de biopsia gástrica de un perro en Japón (GenBank accession number LC090325).

La infección mixta de dos o más especies en forma conjunta no fue un hallazgo frecuente en esta investigación, a diferencia de la mayoría de los demás trabajos, donde lo informan como característico ^9^ . Sólo hubo en una muestra coexistencia de *H. bizzozeronii* y *H. salomonis*.

La alta prevalencia de bacterias NHPH en perros ha generado el interrogante acerca del rol que estos podrían tener en la transmisión al hombre. Si bien no está documentada de manera concluyente la transmisión zoonótica, existen informes que sugieren el pasaje de especies NHPH de los perros a los seres humanos y otros que sustentan el contacto con mascotas de pacientes infectados con especies NHPH.

Aunque en este trabajo no se pudo determinar significancia estadística, quizás debido al pequeño tamaño de la muestra, cuando se analizó la influencia de la presencia de las bacterias en el perro sobre la presencia de bacterias en el humano, el valor OR dio indicios de que la presencia en el perro influiría sobre la presencia en el humano.

Conocer más acerca de las especies de NHPH que colonizan el estómago humano contribuiría a entender la patogénesis, epidemiología y patología de estas infecciones. En tal sentido, es importante el aporte que pueden realizar médicos patólogos y gastroenterólogos profundizando su estudio a través de la aplicación de métodos de diagnóstico más específicos y comunicando el hallazgo de estas bacterias.

## Conclusión

En pacientes con signos clínicos de gastritis crónica, la única especie encontrada fue *H. pylori*.

En sus perros, las especies halladas fueron *H. bizzozeronii*, como la más prevalente, seguida de *H. felis* y *H. salomonis* y por último *H. heilmannii s.s*.

No se encontraron las mismas especies en humanos y sus perros, por lo que no se puede concluir que exista transmisión zoonótica.
